# Hair follicle germs containing vascular endothelial cells for hair regenerative medicine

**DOI:** 10.1038/s41598-020-79722-z

**Published:** 2021-01-12

**Authors:** Tatsuto Kageyama, Yang-Sook Chun, Junji Fukuda

**Affiliations:** 1 Faculty of Engineering, Yokohama National University, 79-5 Tokiwadai, Hodogaya-ku, Yokohama, Kanagawa 240-8501 Japan; 2 Kanagawa Institute of Industrial Science and Technology, 3-2-1 Sakado Takatsu-ku, Kawasaki, Kanagawa 213-0012 Japan; 3 Department of Physiology and Biomedical Sciences, Seoul National University College of Medicine, 103 Daehak-ro, Jongno-gu, Seoul, 110-799 Korea

**Keywords:** Tissue engineering, Regenerative medicine

## Abstract

Hair regenerative medicine has emerged as a promising approach for the treatment of severe hair loss. Recent advances in three-dimensional tissue engineering, such as formation of hair follicle germs (HFGs), have considerably improved hair regeneration after transplantation in animal models. Here, we proposed an approach for fabricating HFGs containing vascular endothelial cells. Epithelial, dermal papilla, and vascular endothelial cells initially formed a single aggregate, which subsequently became a dumbbell-shaped HFG, wherein the vascular endothelial cells localized in the region of dermal papilla cells. The HFGs containing vascular endothelial cells exhibited higher expression of hair morphogenesis-related genes in vitro, along with higher levels of hair shaft regeneration upon transplantation to the dorsal side of nude mice, than those without vascular endothelial cells. The generated hair follicles represented functional characteristics, such as piloerection, as well as morphological characteristics comparable to those of natural hair shafts. This approach may provide a promising strategy for fabricating tissue grafts with higher hair inductivity for hair regenerative medicine.

## Introduction

Hair loss is a common concern worldwide. The International Society of Hair Restoration Surgery had estimated approximately 600,000 patients to have been surgically treated for hair loss worldwide in 2016^1^. Currently, severe androgenetic alopecia is treated primarily by transplantation of the patient’s own hair follicles. However, the outcomes are often unsatisfactory when there are not enough hair follicles available. Furthermore, since this approach only translocates hair follicles from the back of head to the region with sparse hair, the total number of hair follicles does not increase^2^. Therefore, hair regenerative medicine, which in principle increases the number of hair follicles, has emerged as a promising approach for treating alopecia, such as severe androgenetic alopecia, female-pattern hair loss unresponsive to typical drugs, and scarring alopecia.

In vivo development of hair follicles is driven by the formation of hair follicle germs (HFGs) at a location between the epidermal and mesenchymal layers^3^. Replication of such hair follicle development in vitro, including epithelial-mesenchymal interactions, has been considered to engineer tissue grafts for hair regenerative medicine^4–6^. One of the most advanced approaches in this regard is using a bioengineered HFG, prepared by integrating aggregates of epithelial and mesenchymal cells^7^. The bioengineered HFGs efficiently regenerated hair follicles after transplantation in nude mice, which reassembled connections to host arrector pili and nerve fibers. Thus, this approach could partially replicate in vivo hair follicle development. However, since thousands of HFGs are required for a single patient, this approach may be impractical, considering that HFG preparation includes laborious steps involving the handling of individual epithelial and mesenchymal cell aggregates under a microscope. We have recently reported a more straightforward approach for large-scale preparation of HFGs^8,9^. We observed that a mixture of epithelial and mesenchymal cells initially formed an aggregate, where the two types of cells distributed randomly, however, spatially separated from each other following three days of culture, and spontaneously formed HFGs. Due to the self-sorting formation of HFGs, this approach was scalable to fabricate > 5,000 HFGs simultaneously by seeding a suspension of the two cell types in a lab-made microwell array chip. The self-sorted HFGs regenerated hair follicles relatively efficiently, with normal hair cycles, when transplanted into nude mice.

Murine embryonic cells generally exhibit a strong ability to regenerate hair follicles de novo. However, the regenerative ability of adult human hair follicle stem cells often deteriorates even when the same approach is used^10^. Thus, further studies using human cells would be necessary to better replicate in vivo development and hair follicle microenvironment for clinical applications. Perivascular microenvironments are essential for the development and maintenance of stem cells in many tissues. During development, newly forming vessels approach the nascent follicles, and the vascular annulus is completely developed by birth^11^. Reconstituted hair follicles spontaneously form vascular loops around the upper bulge, recapitulating the pattern of normal hair-bearing skin^11^. Furthermore, hair growth and hair follicle size significantly correlate with perifollicular angiogenesis induced by vascular endothelial growth factor^12^. Thus, involvement of vascular endothelial cells in HFGs may be beneficial for replicating hair follicle development and improving de novo hair follicle generation.

**Figure 1:**
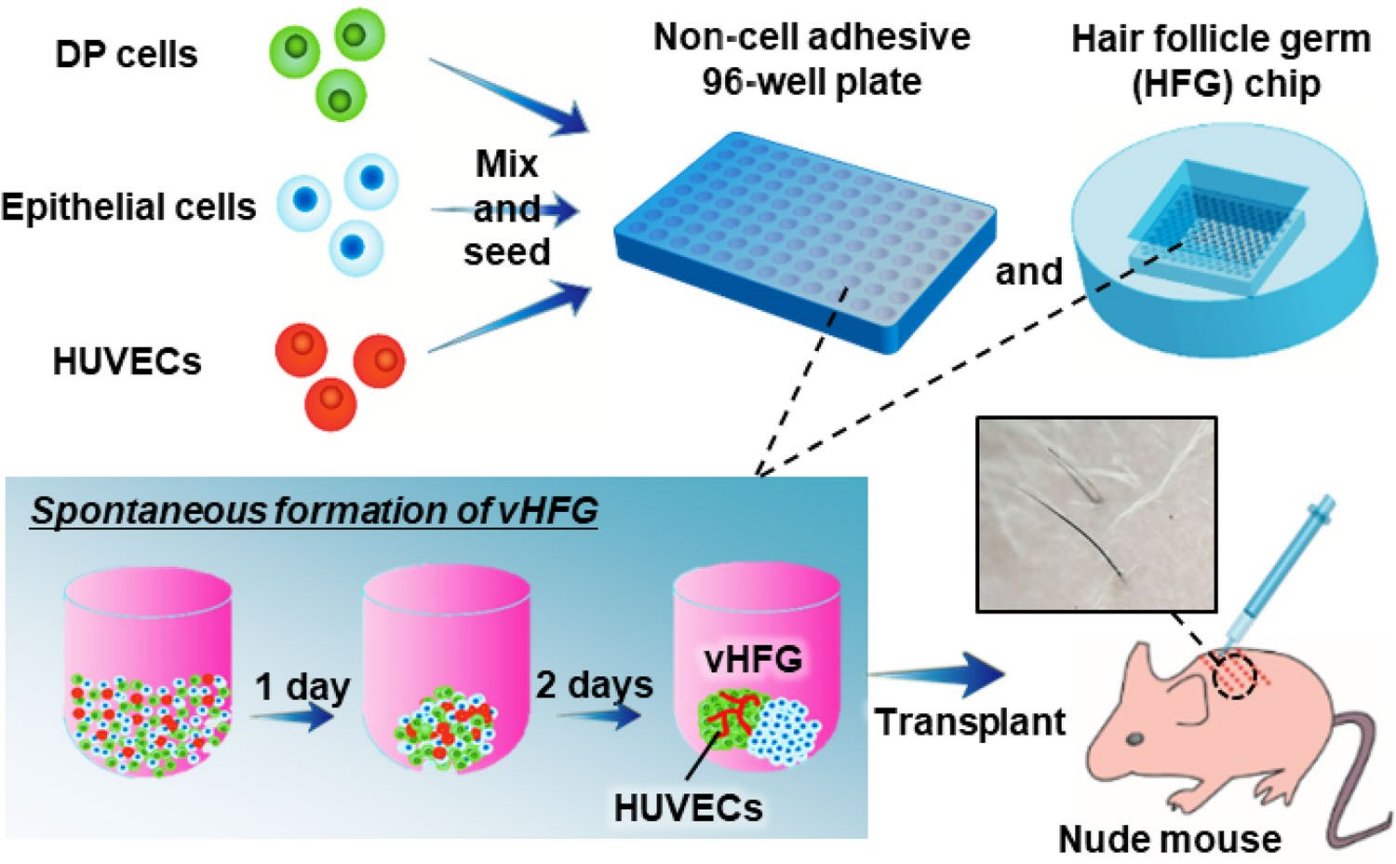
Preparation of vHFGs through self-organization. Human DP cells, murine fetal skin-derived epithelial cells, and HUVECs were mixed and seeded in a non-cell-adhesive 96-well plate and lab-created HFG chip. In each culture vessel, the cells formed single aggregates in the microwells, wherein the three types of cells were randomly distributed after 1 day of culture and spontaneously formed vHFGs in the following 2 days of culture. vHFGs were then transplanted into the dorsal skin of nude mice to evaluate hair regeneration potential.

This study aimed to determine whether vascular endothelial cells participate in the self-organization of HFGs and improve hair regeneration. We added human vascular endothelial cells to a suspension of human dermal papilla (DP) and mouse embryonic epithelial cells and allowed them to form an aggregate in a 96-well plate and custom-designed microwell array plate (Fig. 1). Localization of vascular endothelial cells in an HFG and their effects on follicular gene expression were assessed. Furthermore, we investigated the effects of vascular endothelial cells on hair regeneration in animal models. Our findings provided insight into in vitro self-organization and in vivo morphogenesis of hair follicles, thus providing a promising strategy for hair regenerative medicine.

## Results and discussion

### Self-organization of the three cell types

**Figure 2:**
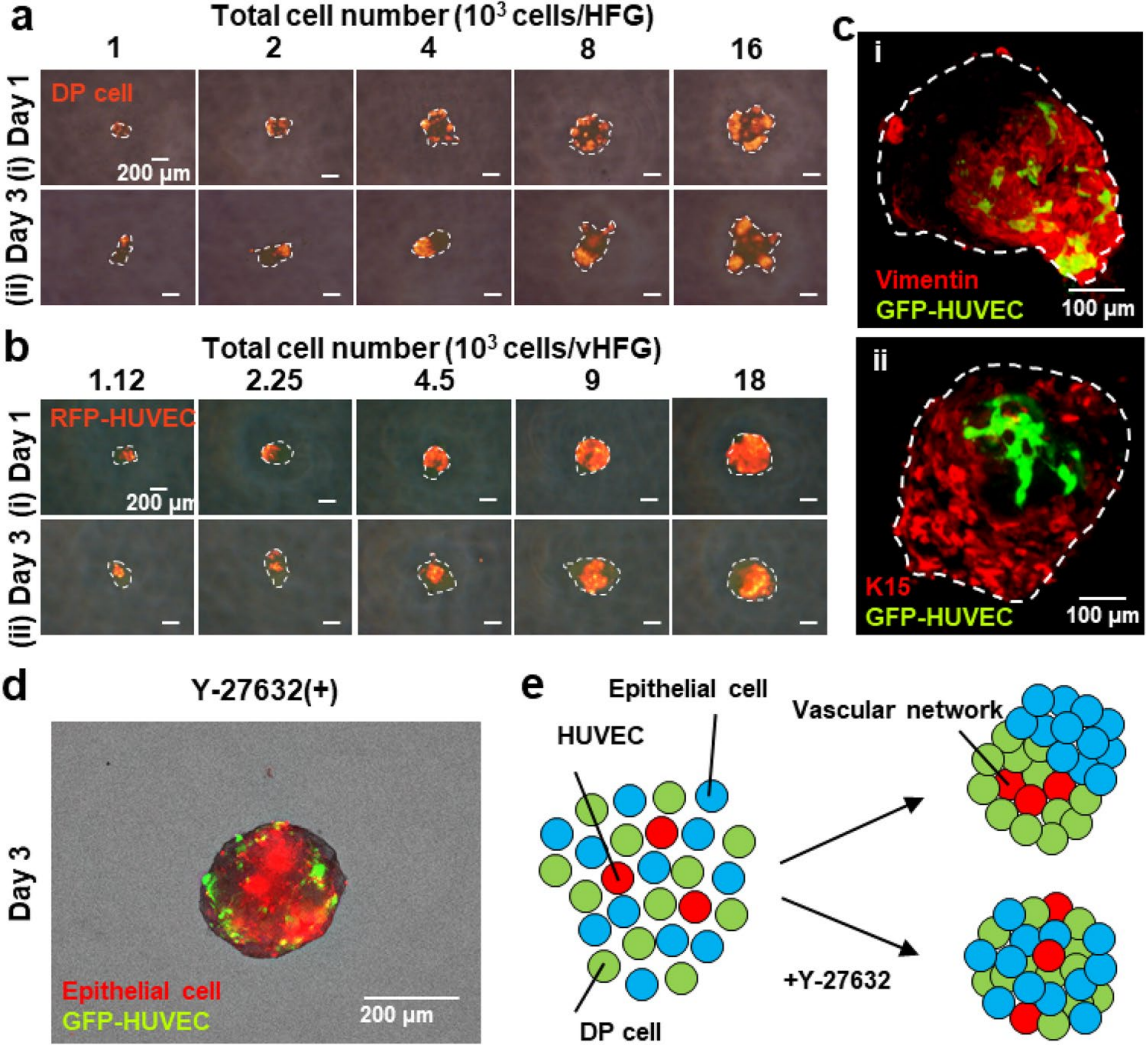
Self-organization of cells into vHFGs. (**a**) Changes in spatial distribution of DP and epithelial cells (1:1 in cell number) in individual aggregates after 1 and 3 days of culture. The DP cells were labeled with fluorescent Vybrant DiI dye before seeding. The fluorescence and phase-contrast images were overlaid. The broken lines indicate the boundary of aggregates. (**b**) Changes in spatial distribution of DP cells, epithelial cells, and HUVECs (4:4:1 in cell number) in individual aggregates at 1 and 3 days of culture. The RFP-HUVECs were used to distinguish them from others. The fluorescence and phase-contrast images are overlaid. The broken lines indicate the boundary of aggregates. (**c**) Confocal microscopic images of immunohistochemically stained vHFGs (9 × 10^3^ cells/HFG). (i) Green, GFP-HUVEC; red, vimentin. (ii) Green, GFP-HUVEC; red, cytokeratin 15 (K15). (**d**) Effects of Y-27632 treatment on self-organization of the three cell types. Red, DiI-labeled mouse epithelial cells; green, GFP-HUVECs. (**e**) Putative schematic of cell sorting behaviors in the presence/absence of Y-27632.

Schematic representation of the experimental procedures is shown in Fig. 1. Note that in vitro proliferation of human hair follicle epithelial stem cells with trichogenic ability has not been established yet, and thus we used mouse embryonic epithelial cells in the current study. Prior to investigation with the three cell types, we examined whether the formation of HFGs through self-sorting of epithelial and mesenchymal cells could occur even among cells from different species. Vybrant DiI-labeled human DP and murine embryonic epithelial cells (1:1) were suspended and seeded in a non-cell-adhesive, round-bottom, 96-well plate at different cell densities (1, 2, 4, 8, 16, 32, or 64 × 10^3^ cells/0.1 mL). During 24 h of culture, the two types of cells formed single aggregates in each well, wherein they were almost randomly distributed and eventually formed small clusters (Fig. 2a (i)). Over the following two days of culture, the cells spatially separated within the individual aggregates and formed HFG-like structures, although DP cells in larger aggregates, composed of > 8 × 10^3^ cells/HFG, did not assemble into a single cluster and rather remained as a few clusters (Fig. 2a (ii)). The dynamic and spatial changes in the chimeric human-mouse HFGs were consistent with the results of our previous study involving HFGs composed of mouse embryonic epithelial and mesenchymal cells, with the exception of larger aggregates^8^. In our previous study, complete separation of the two cell types and formation of dumbbell-like HFGs were observed even with 32 × 10^3^ cells/HFG. Considering the cell sorting mechanisms, these results suggested that human DP cells migrate less and/or show weak homotypic cell–cell adhesion properties.

Human umbilical vein endothelial cells (HUVECs) were added to the suspension of DP and epithelial cells (DP cells: epithelial cells: HUVECs = 4:4:1) to determine their effects on HFG formation in a 96-well plate (Fig. 1). The total number of cells was varied (1.13, 2.25, 4.5, 9, 18, 36, or 72 × 10^3^ cells/well) while maintaining the cell ratio. The number of DP and epithelial cells used here were the same as in the experiments without HUVECs. The addition of HUVECs did not significantly affect cell aggregation or subsequent cell separation during three days of culture (Fig. 2b). Interestingly, HUVECs localized predominantly in the region of DP cells after three days of culture. Confocal laser microscopic analysis also revealed that HUVECs primarily resided in the DP cell region (Fig. 2c). Vimentin is an extracellular matrix component secreted by DP cells while cytokeratin 15 (K15) is an epithelial stem-cell marker^13,14^. The cell aggregate containing HUVECs was designated as vHFG. Similar cell separation and HUVEC localization were observed in vHFGs when the number of HUVECs was doubled (DP cells: epithelial cells: HUVECs = 2:2:1) with different densities (1.25, 2.5, 5, 10, 20, 40, or 80 × 10^3^ cells/well, data not shown). Same results were obtained when a series of different number of murine embryonic mesenchymal cells were used rather than human DP cells (Fig. S1).

Spontaneous vHFG formation may be attributed to the differences in affinities between homotypic and heterotypic intercellular adhesion molecules, as observed in vivo in the mechanisms underlying the morphogenesis of various tissues^15^. Cadherins are typical intercellular adhesion molecules^16,17^. We had previously reported that N- and E-cadherins on mesenchymal and epithelial cells are responsible for HFG formation^8^. Vascular endothelial cells express not only VE-cadherin, but also N-cadherin^18,19^. We confirmed this in HUVECs by immunohistochemical staining of N-cadherin (Fig. S2). To further investigate the mechanism associated with vHFG formation, it was examined in the presence of 30 µM Y-27632, which is a selective inhibitor of the Rho-associated coiled-coil-containing protein kinase (ROCK) that downregulates N-cadherin expression in mesenchymal cells^20^. Results showed that separation of the three cell types was considerably disturbed by the inhibitor (Fig. 2d, e). Thus, the mechanisms underlying vHFG formation, including the localization of HUVECs in DP and mesenchymal cell regions, are likely governed by differences in the cadherin types rather than in the gradients of soluble factors such as oxygen and growth factors.

### Preparation of numerous vHFGs on HFG chip

**Figure 3:**
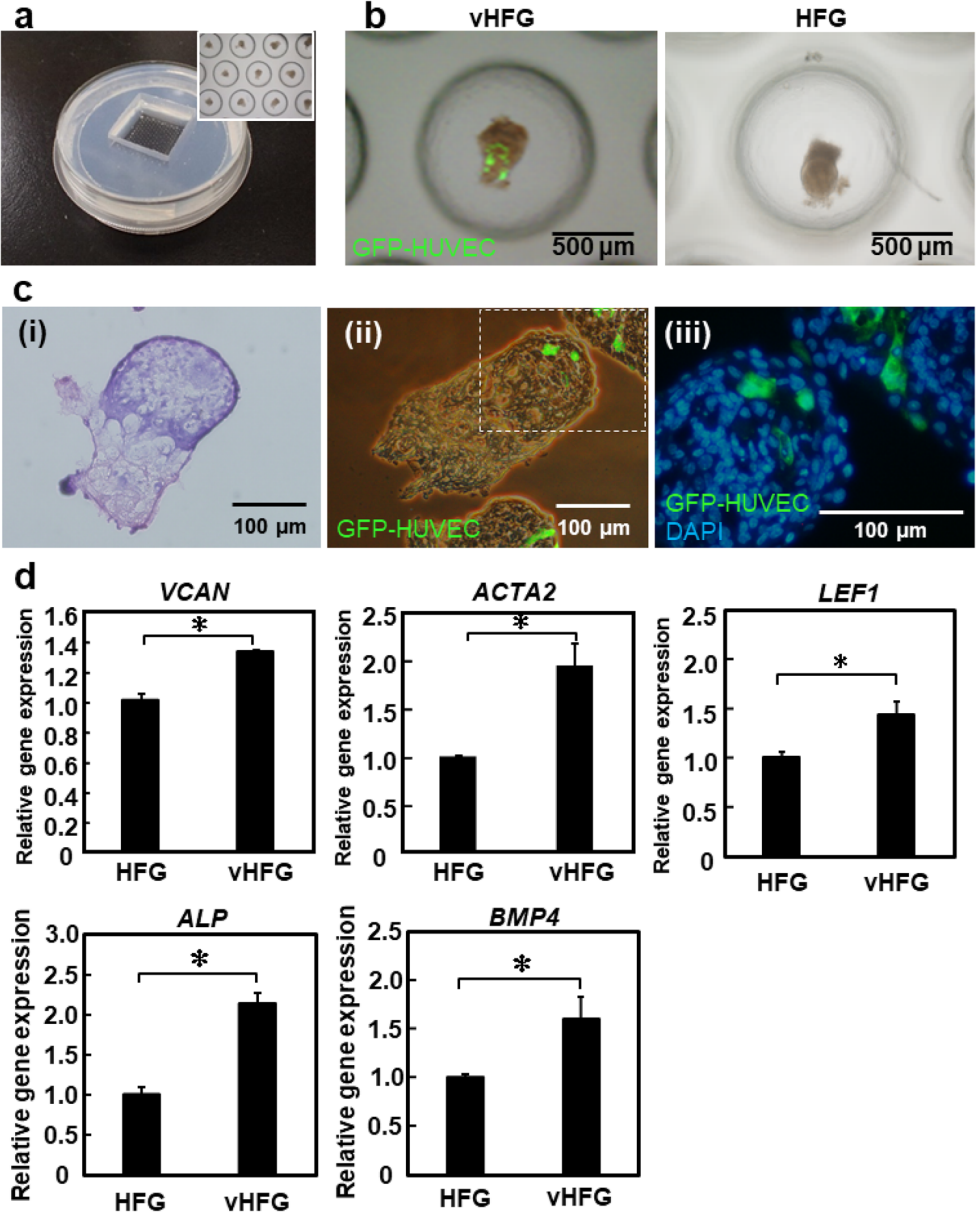
vHFG preparation in HFG chip. (**a**) Fabricated HFG chip. The inset shows a magnified view of vHFGs in microwells. (**b**) Representative appearance of vHFGs and HFGs after 3 days of culture in the HFG chip. The number of cells seeded in the HFG chip was 1.13 × 10^6^ total cells/chip for vHFG and 1 × 10^6^ total cells/chip for HFG. GFP-HUVECs were visualized, and fluorescence and phase-contrast images were overlaid for vHFG. (**c**) HE-staining (i) and fluorescence staining (ii, iii) of cross-sections of vHFGs after 3 days of culture in the HFG chip. GFP-HUVECs (green) and nuclei (blue) were visualized. (**d**) Relative expression of trichogenic genes. *GAPDH* was used as a reference gene to normalize expression. Error bars represent the standard error of mean calculated from three independent experiments for each condition. Variables were statistically evaluated using Student’s *t*-test. *p < 0.05 was considered significant.

We have previously reported a microwell array chip (named as the HFG chip) for the large-scale preparation of HFGs (~ 5,000 HFGs/φ10-cm chip) via self-sorting of epithelial and mesenchymal cells^8^. Since the HFG chip is composed of polydimethylsiloxane (PDMS), an oxygen-permeable silicone, oxygen is supplied not only through the surface of the culture medium, but also through the bottom silicone layer. We predicted that the HFG chip could be used for large-scale preparation of vHFGs, owing to the similar formation processes through self-organization, whereas vascular endothelial cells are generally activated under hypoxia rather than normoxia. Cell suspensions (2 mL) containing DP cells, epithelial cells, and GFP-HUVECs (total density 1.13 × 10^6^ cells/mL, 4:4:1 ratio) were poured into the HFG chips and cultured at 37 °C for three days. As shown in Fig. 3a,b, similar to the results obtained with 96-well plate, DP and epithelial cells were spatially separated from each other, and the HUVECs localized in the DP cell region after three days of culture. Notably, the DP and epithelial cell regions appeared dark and bright, respectively, under a phase-contrast microscope (IX-71, Olympus, Tokyo, Japan), whereas HUVECs were fluorescently labeled in these images. Hematoxylin and eosin (HE) and fluorescence staining of the cross-sections of vHFGs revealed HUVEC localization predominantly in the DP cell region (Fig. 3c). Considering that hair restoration surgery involves the translocation of thousands of hair follicles per patient, a scalable and robust approach for the preparation of thousands of tissue grafts would be essential. Since vHFGs were formed through self-organization, our approach might provide a practical advantage in the large-scale preparation of tissue grafts for hair regenerative medicine.

Previous studies had shown that non-hair follicle cells improve the hair induction functions of hair follicle cells. For example, co-culture of DP cells (as an inner DP core) with adipocyte progenitor cells (as an outer shell) significantly promoted the hair induction ability of DP cells^21^. This was consistent with the fact that the telogen-to-anagen transition is synchronized with differentiation of adipocyte progenitor cells to mature adipocytes near the regenerating hair follicles^22^. Such cell–cell interactions are mediated via direct cell contact and soluble signaling molecules, such as growth factors. Vascular endothelial cells secrete several growth factors, including vascular endothelial growth factor, fibroblast growth factor, and transforming growth factor-β^23,24^. These growth factors are widely used to treat hair loss^12,25,26^. We, therefore, hypothesized that vascular endothelial cells localized in the DP cell region may upregulate follicular genes of DP cells in vHFG. We found that trichogenic DP cell markers, versican *(VCAN)*^27^, alpha-smooth muscle actin 2 *(ACTA2)*^28^, alkaline phosphatase *(ALP*)^29^, bone morphogenetic protein 4 *(BMP4)*^29^, and lymphoid enhancer factor *(LEF1)*^30^, were significantly upregulated in vHFGs compared to HFGs without HUVECs (Fig. 3d). *VCAN* is a well-known hair-inductive marker of DP cells, and HUVECs have also been reported to express *VCAN*^31^. Meanwhile, HUVECs did not significantly affect *WNT5A* or noggin (*NOG)* expression (data not shown). A previous study had reported that DP signature genes, including *NOG*, were upregulated not only by 3D aggregate culture, but also in a synergistic manner with the canonical *WNT* signaling pathway activator CHIR99021^32^. Hence, a similar approach might be adaptable to further improve trichogenic gene expression of DP cells in vHFGs. Although gene expression would be an indicator of hair induction activity, these alone are not thoroughly reliable and animal experiments should be conducted to estimate the activity of hair follicle generation in the skin.

### Comparison between HFGs and vHFGs using the hair-patch assay

**Figure 4:**
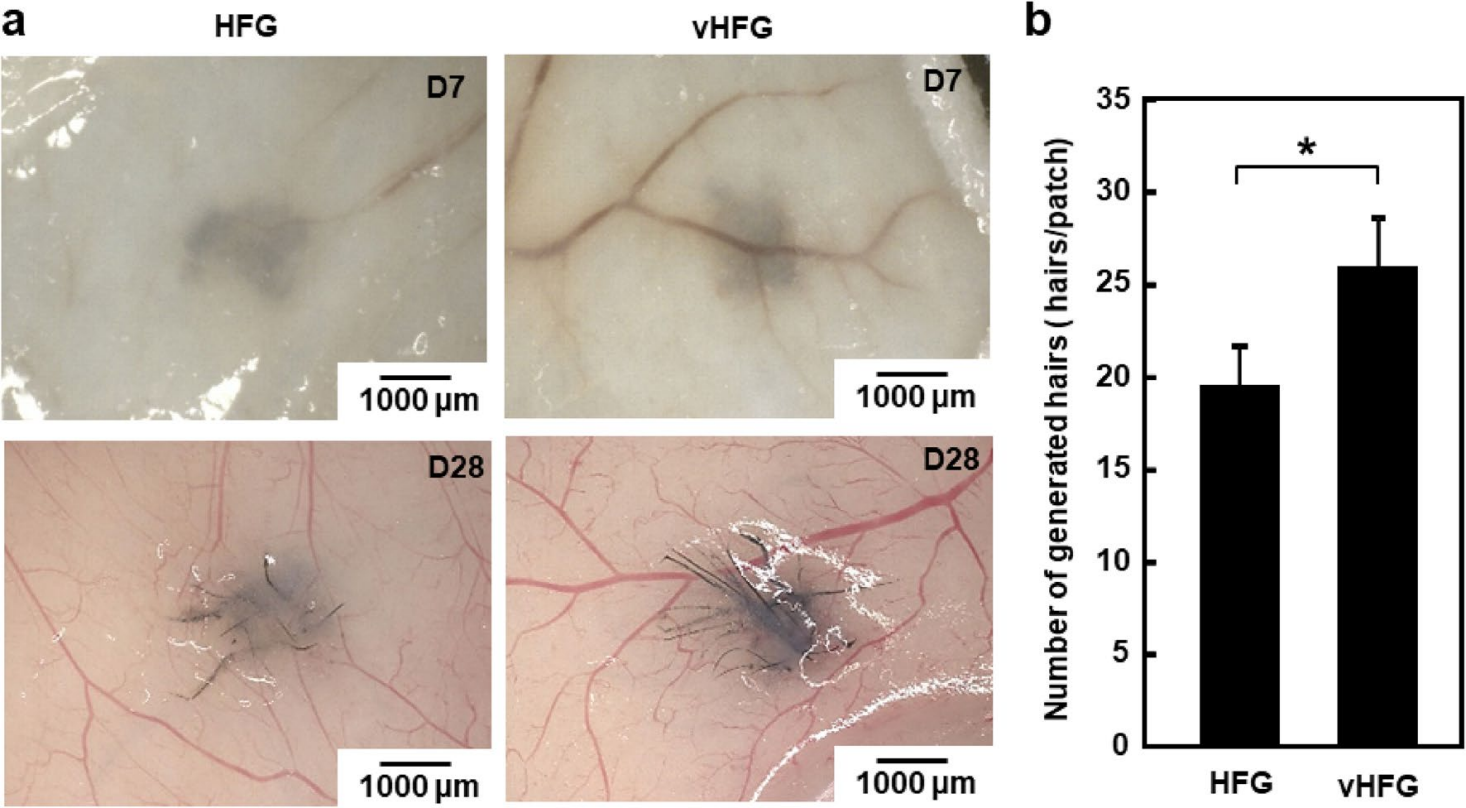
Hair-patch assay with vHFGs. (**a**) Hair shafts generated from 30 vHFGs and 30 HFGs in the lateral dorsal skin. (**b**) Quantification of generated hair shafts. The values and error bars represent at least four experiments for each condition. *p < 0.05 was considered significant. The values and error bars were calculated based on at least five samples.

The ability of vHFGs to generate hair follicles was determined using the hair-patch assay^33^. vHFGs (4.5 × 10^3^ cells/vHFG, 30 aggregates) or HFGs (4 × 10^3^ cells/HFG, 30 aggregates) were transplanted into a wound pocket surgically generated on the lateral dorsal skin of nude mice. Difference in the number of cells between vHFGs and HFGs arose solely from the presence or absence of HUVECs. The vHFG and HFG transplanted sites were pigmented after seven days of transplantation and black hair shafts were generated at all transplanted sites after four weeks of transplantation (Fig. 4a). The clusters of generated hair shafts were extracted after enzymatic dissociation of the tissues and the hair shafts on each transplanted site were counted. The number of hairs generated from vHFGs was significantly higher than that generated from HFGs without HUVECs (Fig. 4b). Recent studies have reported pre-vascularization to significantly improve the engraftment of various three-dimensional tissues^34,35^. Vascular structures in pre-vascularized tissues are typically immature, however, they readily connect to host vasculature and attract blood flow from surrounding tissues to the entire tissue graft before severe depletion of oxygen^34,35^. A technical concern in this study was that, since the epithelial cells were isolated from mouse embryos, possible contamination may have occurred from other cell types, including mesenchymal cells and vascular endothelial cells. To estimate the adverse impact of contamination, spheroids composed of freshly isolated murine epithelial cell suspension were transplanted into the back skin of mice in a similar manner as described for the vHFGs. Three weeks after transplantation, no hair was observed at the transplanted sites (Fig. S4), suggesting that cell contamination was negligible. Although further studies would be necessary to clarify the contributing mechanisms of vascular endothelial cells, the success of engraftment may be attributed to both activation of vHFGs and prompt anastomosis to host vasculature.

### Intracutaneous transplantation of individual vHFGs

Further animal experiments were performed to evaluate in situ hair follicle generation. We initially examined HFGs (without vascular endothelial cells) by grafting them individually into the back skin of nude mice after three days of culture (Fig. S3). To investigate the effect of cell number on hair regeneration, we transplanted HFGs with varying cell numbers into shallow stab wounds generated on the back of mice using a 20-G ophthalmic V-Lance knife (Alcon, Geneva, Switzerland). The transplanted sites were observed every 2–3 days and images of the generated hair were captured using a digital camera (Tough, Olympus). At three weeks after transplantation, HFGs with greater number of cells (> 4 × 10^3^ cells/HFG) generated black hair at the transplanted sites while those with lesser number of cells (< 2 × 10^3^ cells/HFG) showed no hair growth. Hair cycle was observed at approximately 30-day interval (Fig. S3), and was equivalent to that of typical mouse skin^36^. Based on these results, 8 × 10^3^ cells/HFG (DP cells: epithelial cells = 1:1) and 9 × 10^3^ cells/vHFG (DP cells: epithelial cells: HUVECs = 4:4:1) were used for subsequent experiments.

**Figure 5:**
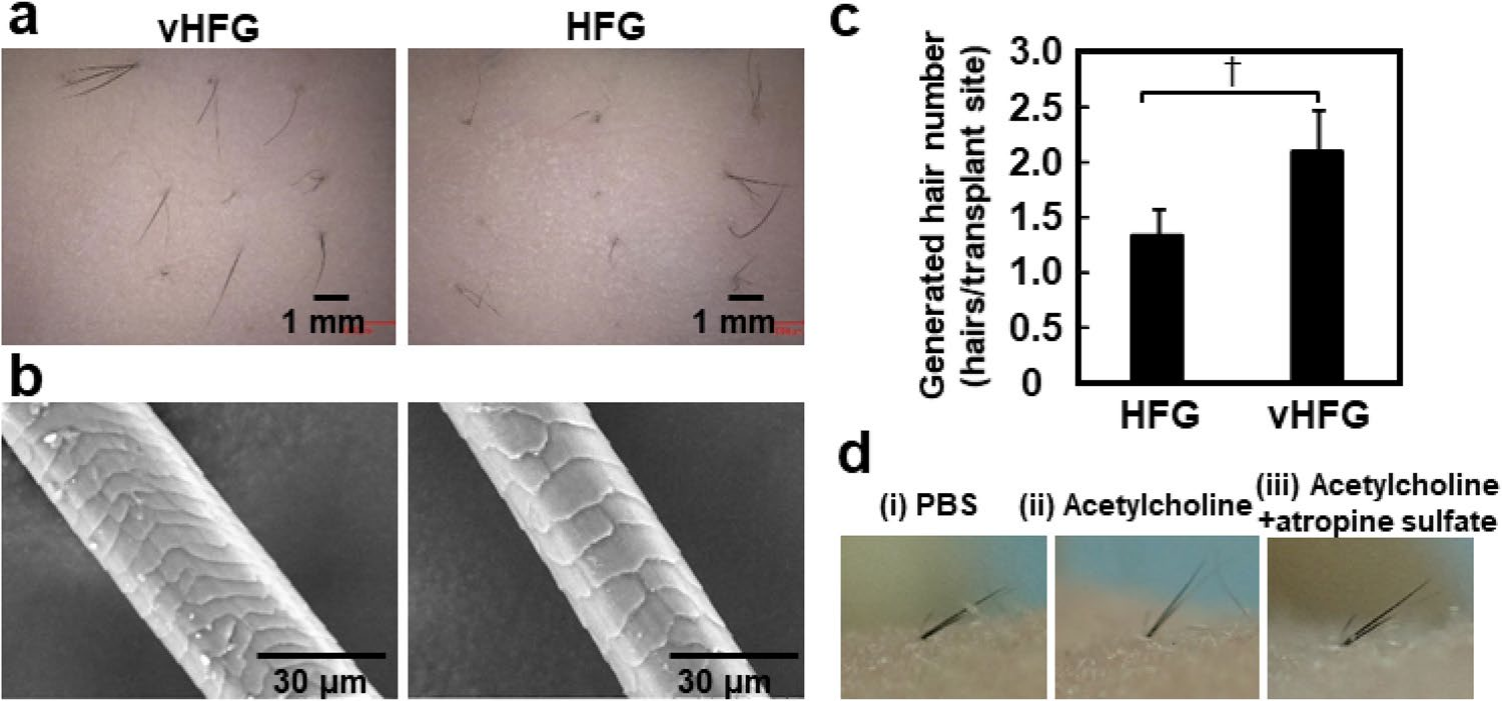
Intracutaneous transplantation of vHFGs and piloerection assay. (**a**) Hair shafts generated 3 weeks after transplantation of vHFGs and HFGs into the dorsal murine skin. (**b**) Scanning electron microscopic images of generated hair shafts. (**c**) Number of generated hair shafts. Values and error bars were calculated from at least 15 HFG transplantations for each condition in three independent experiments. Numerical variables were statistically evaluated using the Student's *t*-test. p < 0.1 is represented by †. (**d**) Piloerection of generated hair shafts after injection of (i) PBS, (ii) acetylcholine, and (iii) acetylcholine plus atropine sulfate.

To compare the hair follicle generation capacity, individual vHFGs (9 × 10^3^ cells/vHFG) or HFGs (8 × 10^3^ cells/HFG) were transplanted into shallow stab wounds generated in mouse dorsal skin. Black hair shafts appeared from the skin surface three weeks after transplantation (Fig. 5a). The hair shafts in both approaches possessed normal morphological characteristics, including the hair cuticle (Fig. 5b). There was no significant difference between vHFGs and HFGs in hair generation efficiency, which was defined as the number of hair-generating sites divided by the number of transplanted sites, whereas the number of generated hairs was higher with vHFGs than with HFGs (Fig. 5c). Piloerection was monitored after intradermal administration of acetylcholine proximal to the generated hair follicles (Fig. 5d). Hair shafts significantly moved vertically after acetylcholine injection, though not after PBS injection (Fig. 5d (i) and (ii). Injection of an anti-cholinergic agent, atropine, suppressed piloerection (Fig. 5d (iii)). Piloerection of regenerated hair shafts was equivalent to that of natural shafts in terms of temporal and spatial characteristics^37^.

This study was inspired by elegant vascular networks surrounding hair follicles in vivo^38^ and by a report that showed prevascularization of an engineered tissue graft containing hair follicle-like structures to significantly improve human hair generation in mice^39^. Although our previous study had demonstrated that > 5000 HFGs could be prepared in the lab-made chip^8^, our approach lacked vascular cells. Our present study demonstrated the significant potential of vascular endothelial cells in the formation of HFGs that would be beneficial for hair regenerative medicine. Although we used accessible cell types as a proof of concept study, cell sources should ideally be of human origin and most preferably autologous to cells in clinical setting. Hence, human DP cells^40^ and dermal sheath cup cells^41^ from patient’s own hair follicles would be the most promising candidates. Regarding epithelial cell sources, hair follicle stem cells located in the bulge region reportedly possess de novo hair generation ability^42^. Since isolation and proliferation of human hair follicle epithelial stem cells while maintaining stemness is a critical issue^43^, iPS-derived epithelial cells could be another cell source^44^. The source of vascular endothelial cells must also be considered. Microvascular endothelial cells surrounding hair follicles, iPS-derived endothelial cells^45^, and endothelial progenitor cell (EPC)-derived endothelial cells^46^ could be used as autologous or immunologically compatible cells.

In summary, we observed localization of HUVECs in the DP cell region in vHFGs. Due to the spontaneous formation of vHFGs, a large number of vHFGs could be prepared by seeding a mixture of DP and epithelial cells, with HUVECs, into a microwell-arrayed HFG chip. The presence of HUVECs upregulated follicular gene expression after three days of culture and significantly increased the number of hairs regenerated after three weeks of transplantation into the dorsal skin of nude mice. The hair shafts possessed piloerection function and showed typical morphological characteristics, such as hair cuticle and medulla. vHFGs may, therefore, be beneficial for developing improved hair regenerative strategies.

## Methods

### Animals

Pregnant C57BL/6 mice were purchased from CLEA, Japan. Five-week-old ICR nu/nu mice were purchased from Charles River, Japan. The animal study was approved by the committee on animal care and use, Yokohama National University (Permit numbers: 2019-04 and 2019-06). Care and handling of mice conformed to the requirements of the above-mentioned committee.

### Preparation of murine epithelial cells

Mouse embryos (E18) were extracted from a pregnant C57BL/6 mouse, and small pieces of their dorsal skin were harvested under a dissecting microscope. Skin tissue was aseptically treated with 4.8 U/mL Dispase II (Sigma Aldrich, St. Louis, MO, USA) for 60 min, and the epithelial and mesenchymal layers were separated using a pair of tweezers^47^. The epithelial layer was then treated with 100 U/mL collagenase type I (Wako, Osaka, Japan) for 80 min and 0.25% trypsin (Thermo Fisher Scientific, Waltham, MA, USA) for 10 min at 37 °C. Debris and tissue aggregates were removed using a 40-μm cell strainer (Corning, Corning, NY, USA). After centrifugation at 200 × *g* for 3 min, the epithelial cells were suspended in KG2 (Kurabo, Osaka, Japan) and counted.

### Preparation of human DP cells and HUVECs

Human DP cells (PromoCell, Heidelberg, Germany) were maintained in follicle DP cell growth medium (DPCGM) (PromoCell), which was changed every 2–3 days. Cells from the fourth passage were used in the experiments. HUVECs (Angio-proteomie, Boston, MA, USA) and GFP/RFP-induced HUVECs (GFP/RFP-HUVEC, Angio-proteomie) were maintained in endothelial cell growth medium (EGM-2; Lonza, Basel, Switzerland), which was refreshed every 2–3 days. Cells from passages 5–7 were used in the experiments. Mixed culture medium, composed of DPCGM, KG2, and EGM-2 (1:1:1 ratio), was used for HFG culture in the HFG chips.

### Fabrication of HFG chips

HFG chips were fabricated via micro-milling and molding, as described previously^8^. Briefly, configuration of the microwell arrays (diameter, 1 mm; pitch, 1.3 mm; depth, 500 µm) in a 20 × 20-mm region (250 microwells) was designed using CAD/CAM software (VCarve pro, Vectric, Alcester, UK). The design data were subsequently sent to a computer-aided micro-milling machine (MDX-540, Roland, Irvine, CA, USA) to fabricate a negative mold in polyolefin plate (ZEONOR, Zeon, Tokyo, Japan). Epoxy resin (Nissin Resin, Tokyo, Japan) was then poured on the negative mold and cured at room temperature for 24 h to produce a positive mold. The PDMS solution (Shin-Etsu Silicones, Tokyo, Japan) (prepolymer solution and curing agent were mixed in 10:1 ratio) was poured onto the positive mold and cured in an oven at 80 °C for 3 h. Thickness of the PDMS substrate at the bottom of the microwells, which was responsible for supplying oxygen to the culture, was set to 1.5 mm by adjusting the PDMS volume. The PDMS substrate was immersed in pure water and autoclaved for sterilization. Surface of the PDMS substrate and microwells were treated with 4% pluronic F-127 (Wako) solution for 12 h to prevent cell adhesion onto the surface. After substantial washing with PBS to remove excess pluronic F-127, the PDMS substrate was used for cell culture.

### Human-mouse chimeric HFGs containing HUVECs (vHFG)

To prepare HFGs, DP and epithelial cells were suspended in 0.1 mL culture medium (1, 2, 4, 8, 16, 32, or 64 × 10^3^ cells/0.1 mL; DP cells: epithelial cells = 1:1). To prepare vHFGs, RFP/GFP-HUVECs, DP cells, and epithelial cells were suspended in 0.1 mL EGM2/DMEM/KG2 (1.13, 2.25, 4.5, 9, 18, 36, or 72 × 10^3^ cells/0.1 mL, DP cells: epithelial cells: RFP-HUVEC = 4:4:1; 1.25, 2.5, 5, 10, 20, 40, or 80 × 10^3^ cells/0.1 mL, DP cells: epithelial cells: RFP-HUVEC = 2:2:1). Suspensions containing the three cell types were mixed and seeded in wells of a non-cell-adhesive, round-bottom, 96-well plate (prime surface 96U; Sumitomo Bakelite, Tokyo, Japan). After 3 days of culture, spatial distribution of the cells was examined under a confocal microscope (LSM 700, Carl Zeiss, Oberkochen, Germany) and a fluorescence microscope (DP-71, Olympus).

### Large-scale preparation of vHFGs

Cell suspensions (2 mL) containing DP cells, epithelial cells, and GFP-HUVECs (total density 1.13 × 10^6^ cells/mL, 4:4:1 ratio) were poured into the HFG chips and cultured at 37 °C in mixed culture medium composed of DPCGM, KG2, and EGM-2 (1:1:1 ratio) for three days. Spatial distribution of the cells was examined after three days using a phase-contrast microscope (IX-71, Olympus) and fluorescence microscope (DP-71, Olympus), and the relative expression levels of genes associated with hair follicle induction were assessed by real-time reverse-transcription polymerase chain reaction (RT-PCR).

### Gene expression analysis using RT-PCR

Total RNA was extracted from samples using an RNeasy mini-kit (Qiagen, Hilden, Germany), and cDNA was synthesized by reverse-transcription using a ReverTraAce RT-qPCR kit (Toyobo, Osaka, Japan), according to the manufacturer's instructions. qPCR was performed using the StepOne Plus RT-PCR system (Applied Biosystems, Foster City, CA, USA) and SYBR Premix Ex Taq II (Takara-bio, Shiga, Japan). Gene expression was assessed using primers for the following genes: *VCAN* (versican), 5ʹ-CCAGCAAGCACAAAATTTCA-3ʹ and 5ʹ-TGCACTGGATCTGTTTCTTCA-3ʹ; *ACTA2* (α-smooth muscle actin, α-SMA), 5ʹ-GCTTCCCTGAACACCACCCAGT-3ʹ and 5ʹ-GCCTTACAGAGCCCAGAGCCAT-3ʹ; *LEF1* (lymphoid enhancer-binding factor 1), 5ʹ-CCCGATGACGGAAAGCAT-3ʹ and 5ʹ-TCGAGTAGGAGGGTCCCTTGT-3ʹ; *ALP* (alkaline phosphatase), 5ʹ-ATTGACCACGGGCACCAT-3ʹ and 5ʹ-CTCCACCGCCTCATGCA-3ʹ; *BMP4* (bone morphogenetic protein 4), 5ʹ-GCCCGCAGCCTAGCAA-3ʹ and 5ʹ-CGGTAAAGATCCCGCATGTAG-3ʹ; and *GAPDH* (glyceraldehyde-3-phosphate dehydrogenase), 5ʹ-GCACCGTCAAGGCTGAGAAC-3ʹ and 5ʹ-TGGTGAAGACGCCAGTGGA-3ʹ. Expression levels of all the genes were normalized to that of *GAPDH*. Relative gene expression was determined using the 2^−⊿⊿Ct^ method and presented as the mean ± standard error of four independent experiments. Statistical analysis of variables was conducted using Student's *t*-tests, and differences with p < 0.05 were considered statistically significant.

### Hair-patch assay

The hair induction ability of vHFGs was quantified using the hair-patch assay^48^. Briefly, under isoflurane anesthesia, a wound (4–6 mm in diameter) was surgically generated on the lateral dorsal skin of mice. The vHFGs (4.5 × 10^3^ cells/vHFG, 30 aggregates) and HFGs (4 × 10^3^ cells/HFG, 30 aggregates) were transplanted separately into the wound. At four weeks after transplantation, all transplanted sites were examined using a digital microscope (VHX-1000, Keyence, Osaka, Japan) and digital camera (Tough, Olympus). To dissociate the generated hair shafts from the skin, a piece of skin (~ 1 ×  ~ 1 cm) was treated with 100 U/mL collagenase at 37 °C for 2 h. The number of hair shafts per transplanted site was counted after hair dissociation.

### Intracutaneous transplantation

The mice used in these experiments were raised under specific pathogen-free conditions and had access to chow and water ad libitum. Under isoflurane anesthesia, HFGs and vHFGs were transplanted into the shallow stab wounds generated on the back of ICR nu/nu mice using a 20-G ophthalmic V-Lance knife (Alcon). The transplantation sites were then rubbed with ointment and observed every 2–3 days, and images of the generated hairs were captured using a digital camera (Tough, Olympus). Cuticles of generated hairs were examined using a scanning electron microscope at 5–10 kV without any treatment.

### Piloerection of regenerated hairs

Piloerection was assessed after injecting the mice with neurotransmitters, as described previously^37^. PBS (1 µL) was injected to prevent possible cutaneous reflex movements. Subsequently, 1 µL of 10 mg/mL acetylcholine solution (Sigma Aldrich) was injected intradermally near the regenerated hair follicles. To determine the specificity of acetylcholine for initiating piloerection, 1 µL of a 100 mg/mL solution of atropine sulfate (acetylcholine inhibitor; Sigma Aldrich) was injected before acetylcholine injection. The angles of piloerection were recorded using a digital camera (Tough, Olympus).

### Histological and immunohistochemical staining

Samples were washed with PBS and fixed with 4% formaldehyde (Sigma Aldrich) for 1 h at room temperature. After embedding in a paraffin block, 8-μm-thick sections were cut and stained with Meyer’s hematoxylin and eosin Y solutions (Muto Pure Chemicals, Tokyo, Japan). For immunohistochemical staining, the sections were fixed in 10% formaldehyde (pH 7) overnight at room temperature. The samples were first blocked in PBS containing 1% bovine serum albumin (Thermo Fisher Scientific) and 0.01% Triton X-100 (Thermo Fisher Scientific) for 1 h at room temperature and subsequently incubated overnight with anti-vimentin (rabbit, primary antibody, Merck Millipore, Billerica, MA, USA) and anti-cytokeratin 15 (rabbit, primary antibody, Abcam, Cambridge, UK) at 4 °C. The sections were incubated with the corresponding secondary antibodies (highly cross-absorbed goat anti-IgG (H + L) Alexa Fluor 488 or goat anti-IgG (H + L) Alexa Fluor 555, Thermo Fisher Scientific) in blocking solution for 3 h at room temperature, and finally stained with DAPI (Sigma Aldrich) in PBS for 8 min. A confocal microscope (LSM 700; Carl Zeiss) was used for fluorescence imaging.

## Supplementary information

Supplementary Figures.
