# Supplementary material for: Hair follicle germs containing vascular endothelial cells for hair regenerative medicine
Source: Sci Rep. 2021 Jan 12;11:624. doi: 10.1038/s41598-020-79722-z (PMC7804392; doi:10.1038/s41598-020-79722-z)
Supplement: Supplementary file 1 — Supplementary Figures. [file 41598_2020_79722_MOESM1_ESM.docx]

**Supplementary information**

**Hair follicle germs containing vascular endothelial cells for hair regenerative medicine**

Tatsuto Kageyama^a, b^, Yang-Sook Chun^c^ and Junji Fukuda^a, b^*

^a^Faculty of Engineering, Yokohama National University, 79-5 Tokiwadai, Hodogaya-ku, Yokohama, Kanagawa 240-8501, Japan

^b^Kanagawa Institute of Industrial Science and Technology, 3-2-1 Sakado Takatsu-ku, Kawasaki, Kanagawa 213-0012, Japan

^c^Department of Physiology and Biomedical Sciences, Seoul National University College of Medicine, 103 Daehak-ro, Jongno-gu, Seoul 110-799, Korea

**
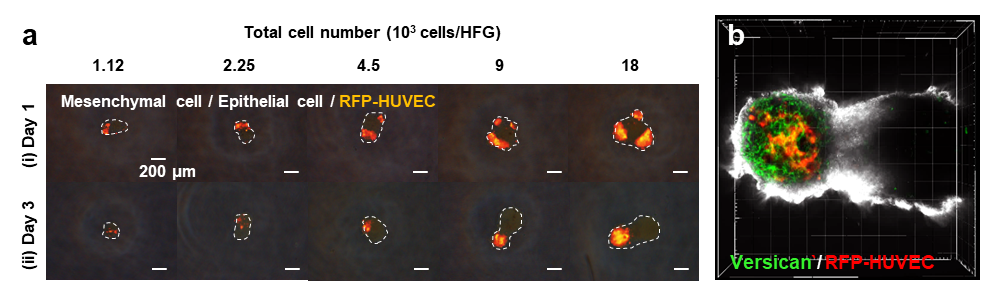
**

**Fig. S1. vHFGs consisting of murine embryonic mesenchymal and epithelial cells, and HUVECs.** (a) vHFG composed of different total cell numbers (mesenchymal cells: epithelial cells: HUVECs = 4:4:1). RFP-HUVECs were used to distinguish from the others. The fluorescent- and phase-contrast images were overlaid. The broken lines indicate the boundary of the aggregates. (b) Confocal microscopic images of vHFG (seeding cell number, 9 × 10^3^ cells/HFG). Green, versican; red, RFP-HUVECs.


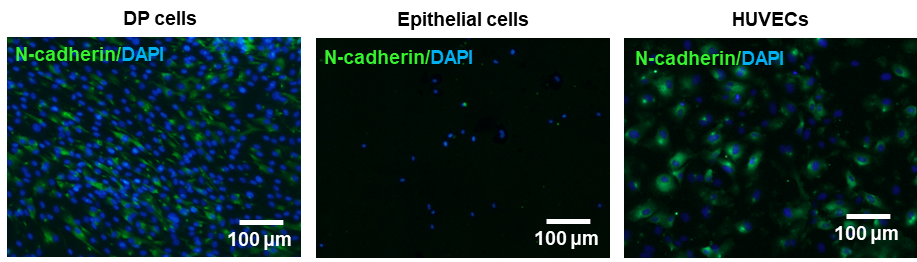


**Fig. S2. N-cadherin expression on DP, epithelial cells, and HUVECs.** N-cadherin expressed on DP cells and HUVECs, but not on epithelial cells. Green, N-cadherin; blue, nuclei.


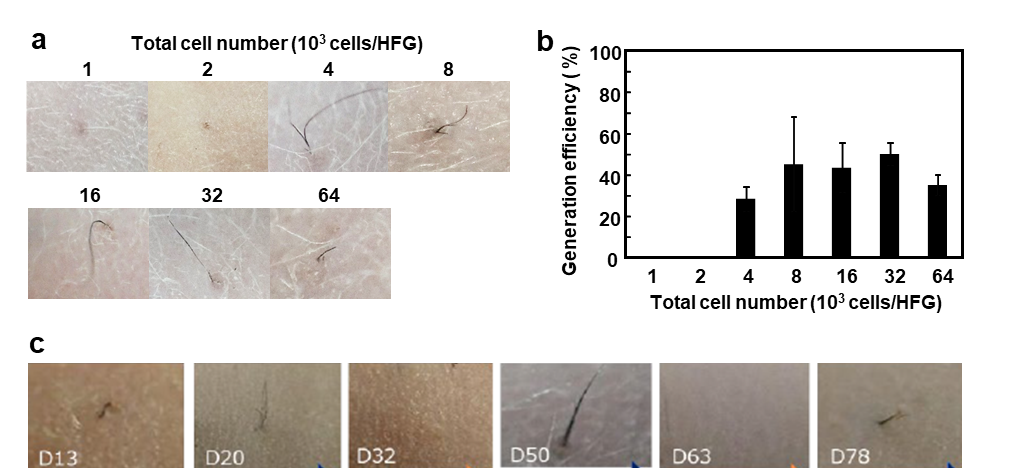


**Fig. S3. Hair shaft generation using chimeric human–mouse HFGs composed of varying numbers of cells**. (a) Stereomicroscopic images of murine back skin 3 weeks after transplantation of HFGs prepared with the indicated number of cells. (b) Efficiency of hair shaft generation. The generation efficiency was defined as the ratio of the number of hair generated sites to the total number of transplanted sites. For each cell number, three independent replicates of at least 10 HFGs were transplanted. Error bars represent the standard error calculated from three independent experiments. (c) Hair cycle. Repeated hair shaft generation was observed at the same location of mouse skin at the indicated day after transplantation.


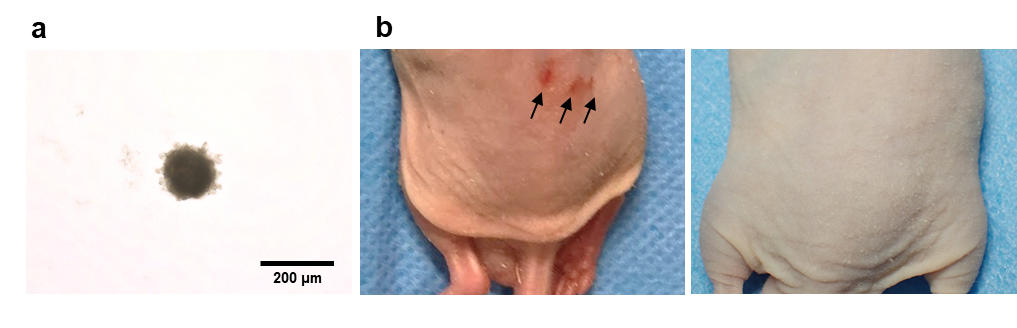


**Fig. S4. Epithelial cell aggregate and transplantation onto mice.** (a) Epithelial cell spheroids cultured for three days. Mouse epithelial cells (8 × 10^3^ cells/well) were suspended in KG2/DPCGM/EGM-2 medium and cultured in 96 well plate. (b) Transplantation of epithelial cell aggregates. Epithelial cell spheroids were transplanted into the shallow stab wounds (arrows) generated on the back of mice using a 20-G ophthalmic V-Lance knife. The transplantation sites were captured using a digital camera at day 0 (left) and three weeks (right) after transplantation.
